# Prediction and Utilization of Malondialdehyde in Exotic Pine Under Drought Stress Using Near-Infrared Spectroscopy

**DOI:** 10.3389/fpls.2021.735275

**Published:** 2021-10-18

**Authors:** Yini Zhang, Qifu Luan, Jingmin Jiang, Yanjie Li

**Affiliations:** Research Institute of Subtropical Forestry, Chinese Academy of Forestry, Fuyang, China

**Keywords:** model calibration, abiotic stress, NIR spectroscopy, non-destructive, pine tree

## Abstract

Drought is a major abiotic stress that adversely affects the growth and productivity of plants. Malondialdehyde (MDA), a substance produced by membrane lipids in response to reactive oxygen species (ROS), can be used as a drought indicator to evaluate the degree of plasma membrane damage and the ability of plants to drought stress tolerance. Still measuring MDA is usually a labor- and time-consuming task. In this study, near-infrared (NIR) spectroscopy combined with partial least squares (PLS) was used to obtain rapid and high-throughput measurements of MDA, and the application of this technique to plant drought stress experiments was also investigated. Two exotic conifer tree species, namely, slash pine (*Pinus elliottii*) and loblolly pine (*Pinus taeda*), were used as plant material exposed to drought stress; different types of spectral preprocessing methods and important feature-selection algorithms were applied to the PLS model to calibrate it and obtain the best MDA-predicting model. The results show that the best PLS model is established *via* the combined treatment of detrended variable–significant multivariate correlation algorithm (DET-sMC), where latent variables (LVs) were 6. This model has a respectable predictive capability, with a correlation coefficient (*R*^2^) of 0.66, a root mean square error (RMSE) of 2.28%, and a residual prediction deviation (RPD) of 1.51, and it was successfully implemented in drought stress experiments as a reliable and non-destructive method to detect the MDA content in real time.

## Introduction

Slash pine (*Pinus elliottii*) and loblolly pine (*Pinus taeda*) trees were introduced to China in the past century, and due to their high-resin yield, fast growth, and suitably long humid, warm-temperate climatic condition, they are mostly cultivated in the south of the country (Yi et al., 2000; Liu et al., 2013; Lilian et al., 2021). In recent years, both slash and loblolly pine have attained key status in terms of their ecological and economic benefits. The annual yield of resin in China is ca. 0.6 million tons, accounting for 50% of the global turpentine trade (Acosta et al., 2019; McConnell et al., 2021; Yi et al., 2021). However, resin yield and wood quality are affected by biological and abiotic stresses (Towler et al., 2015), and understanding the degree of important stress factors and taking timely measures to effectively avoid their adverse effects on turpentine yield and wood properties is imperative.

Plants incur lipid peroxidation in response to oxidative stress after encountering various forms of adversity or when they undergo senescence, which leads to the destruction and protein lysis of the cell membrane system, thereby impairing plant photosynthesis and respiration, causing the death of plant cells in severe cases (Janku et al., 2019). Malondialdehyde (MDA) is one of the final products of polyunsaturated fatty acid peroxidation in the cells; for this reason, it is a widely used and reliable marker for determining the degree of injury to a stressed plant (Morales and Munné-Bosch, 2019). The more the plant is damaged, the higher its MDA content, as found in studies that focused on plant responses to abiotic and biotic stresses (Alché, 2019). That is to say, plants will generate ROS under abiotic or biotic stress conditions, thereby impairing the production of biomolecules, such as lipids, proteins, and nucleic acids, which increases the MDA content and the permeability of the plasma membrane, leading to extravasation of the content of cells. It is the mechanism by which drought resistance in plants is regulated (Munnik et al., 2000; Kong et al., 2016). Therefore, the MDA content could be used as a robust diagnostic indicator when studying plant growth dynamics, such that predicting the MDA content in plants could be used to know the stress conditions of plants in real-time, enabling corresponding pre-emptive measures against drought to be taken. Still, our understanding of methods and results in membrane lipid peroxidation markers remains limited by several shortcomings (Munnik et al., 2000).

The thiobarbituric acid (TBA) assay has been the most common method used for determining MDA in plants. This mainly relies on the chromogenic reaction of TBA with MDA under acidic conditions to produce reddish-brown 3,5,5-trimethyloxazole-2,4-ditone (C_6_H_9_NO_3_), whose absorbance values at 532- and 600-nm wavelengths are compared to calculate the MDA content (Janero, 1990; Hodges et al., 1999). However, this method is susceptible to interference from carbohydrates in plants, which affects the final measurement results (Xu et al., 1993). Furthermore, traditional methods devised to empirically determine these indicators are time-consuming, laborious, and destructive. Hence, it is of great significance to establish a rapid, effective, non-destructive, and non-polluting way to determine MDA for drought resistance of plants.

Near-infrared (NIR) spectroscopy is a highly flexible form of analysis, whose applications began in the 1950s (Pasquini and Celio, 2003; Mohamed et al., 2018; Chu et al., 2020). Since then, through over half a century of development, it has matured and is now widely employed in food, medicine, petrochemical, and other research fields (Mohamed et al., 2018; Guo et al., 2020). In recent years, NIR spectroscopy technology has become increasingly and broadly used in forestry, for example, to estimate photosynthetic characteristics (Dechant et al., 2017), to predict leaf-level nitrogen content (Kokaly, 2001), to distinguish bamboo shoots of different qualities (Tong et al., 2020), and to name a few applications. Partial least squares (PLS) regression, a quick, efficient, and optimal regression method based on covariance, is a widely used chemometric method, one that combines the advantages of multiple linear regression, canonical correlation analysis, and principal component regression (Tenenhaus et al., 2005; Sarker and Nichol, 2011). This approach has been applied to rapidly predict leaf photosynthetic parameters (Meacham et al., 2019), analyze the quantitative of forest biomass (Acquah et al., 2016), among others. It is worth noting that substantial spectral data will contain redundant and complicated information. Therefore, to establish a moderately practical model, it is necessary to preprocess the collected spectral data (Xu et al., 2008).

Preprocessing of NIR spectral data has become a crucial step in chemometric modeling. The target of this preprocessing is to remove physical phenomena, including a specific source of noise and overlapping information, from the spectra so as to improve the subsequent multivariate regression, classification model, or exploratory analysis (Rinnan et al., 2009). Similarly, variable selection is also a critical step in spectral analysis, which can select the most relevant spectral band to improve the overall performance of the model (Yu et al., 2020). Surprisingly, the prediction of MDA in *P. elliottii* and *P. taeda* under drought stress has yet to be reported.

Therefore, this study aimed (1) to derive a technique to reliably estimate and predict MDA of *P. elliottii* and *P. taeda* under different drought stress conditions using NIR technology; (2) to compare the performance of different preprocessing methods and differing feature-variable selection methods; and (3) to evaluate the response of leaf-level MDA in *P. elliottii* and *P. taeda* under drought stress using the NIR-based technique.

## Materials and Methods

### Site and Plants

The experimental site was a greenhouse at the Research Institute of Subtropical Forestry, Chinese Academy of Forestry, located in Hangzhou, Zhejiang Province, China (30°3′N, 119°57′E).

The experiment materials consisted of 1-year-old container seedlings of slash pine and loblolly pine. Watering, fertilizing, and other managements were all implemented according to the growth requirements of both species. The drought stress treatment began after 3 weeks of acclimatization of seedlings to the greenhouse conditions. Two experiments were set up for model calibration and drought stress investigation. In each experiment, the watering regime was set during the seedling tempering period as the normal watering amount (control check), and this watering amount was then reduced into four different drought stress conditions as follows: by 20% (treatment 1), 40% (treatment 2), 60% (treatment 3), and 80% (treatment 4). There are 15 biological replicates per species under each treatment, and in total, 150 samples per experiment were used.

### NIR Spectrum Measurements

Near-infrared spectral data were collected in August and September 2020 using a field-based spectrometer (LF-2500, Spectral evolution, USA). For each scan, the fresh needle samples were arranged tightly to minimize other noise pollution, placed on a background board, and scanned directly with a handheld fiber optic contact probe; spectra were averaged after 20 scans per sample, whose values ranged from 1,000 to 2,500 nm with a 6-nm resolution. (1) For the model calibration experiment, three samples per treatment were selected for NIR data collection and MDA content measurement on days 0, 7, 14, 21, and 27 at 9:00–10:00 a.m. (2) For the same drought stress experiment, all the 150 samples have only been taken NIR spectra on the same day when the NIR data were taken in the model calibration experiment.

### MDA Measurement of Conifers

A commercially available detection kit for MDA measurement was used (Suzhou Keming Biotechnology Company). For this, mixed fresh needles were weighed to ca. 0.1 g and then ground into powder with a 1-ml extracting solution. The extraction was centrifuged for 10 min at 8,000 *rcf* at 4°C. Then, 0.2 ml of the supernate was removed and mixed with 0.6 ml of TBA and moderately shaken. These mixed liquids were placed in a 95°C water bath for 30 min and then centrifuged for 10 min at 10,000 *rcf* at 25°C. Each extraction was placed in a spectrophotometer, and its recorded absorption at 532 and 600 nm wavelengths were used to determine the MDA concentration of that sample (Chen and Wang, 2012).

### Preprocessing and Variable Selection of NIR

To reduce bias from physical factors and irrelevant variables on the establishment of a stable and reliable model (Liang et al., 2020), six preprocessing methods, namely standard normal variate (SNV), detrended variable (DET, *p* = 2), block scale (BS), block normal (BN), block scale and standard normal variate (BS-SNV), and detrended variable and standard normal variate (DET-SNV), were combined with PLS (Wold et al., 2001). Four variable selection methods were applied as follows: inverse variable elimination (bve) (Eason, 1990), genetic algorithm (ga) (Molajou et al., 2021), regularization elimination (rep) (Mehmood et al., 2012; Molajou et al., 2021), and a significant multivariate correlation (sMC) algorithm (Tran et al., 2014). The calibration set (*n* = 80 samples) was used to develop a calibrated model, and the separate validation set (*n* = 20 samples) was reserved to assess and evaluate the prediction performance of the developed model. Three indicators of internal cross-validation, namely, the correlation coefficient (*R*^2^), root mean square error (RMSE), and residual prediction deviation (RPD), were used to assess model robustness. For that, the closer the *R*^2^ to 1 and the RMSE to 0 and higher values for the RPD, the better the prediction ability of the model is (Yan et al., 2000; Plans et al., 2012). Finally, the optimum number of PLS components (latent variables (LVs)) were selected.

### Software Tools

All the data analyses were implemented using R software (v4.0.4). The “pls” (Mevik and Wehrens, 2007) and “enpls” (Xiao et al., 2019) packages of R were used for building the PLS model, and the “prospectr” package (Stevens and Ramirez-Lopez, 2020) was used for manipulation of the NIR spectral data, with the “ggplot2” (Wickham, 2017) package for drawing the plots.

## Results

### Model Performance

Six spectral preprocessing methods and four kinds of variable selection were used for model calibration, whose results are shown in Table 1. Compared with the model without data processing, the accuracy of the model established was improved when using the SNV, DET, DET-SNV, and BS-SNV spectral preprocessing methods. For all models, the average *R*^2^ and RMSE values of the calibration and validation sets were 0.64 (range: 0.63–0.65), 2.30% (range: 2.28–2.33%) and 0.61 (range: 0.61–0.62), 2.98% (range: 2.96–2.99%) respectively. In addition, the average RPD was 1.31 (range: 1.04–1.51). Despite the variable selection methods, the DET processing method yielded the highest accuracy, with *R*^2^ and RMSE values for the calibration set of 0.65 (range: 0.65–0.66) and 2.28% (range: 2.26%−2.28), respectively, and the values of RPD was 1.40 (range: 1.35–1.51). Then, performance was ranked in the following order: DET-SNV, BS-SNV, SNV, BS, and original spectrum (OG) processing methods, followed by BN showing the worst accuracy whose mean values for *R*^2^ and RMSE were 0.64 (range: 0.63–0.64), 2.32% (range: 2.31–2.33%), and 0.61 and 2.97% (range: 2.96–2.98%) for the calibration and validation sets, respectively. Compared with the full spectrum PLS model, the ga, rep, and sMC variable selection methods generated similar results when applied to all preprocessing model types. Among these, the sMC variable selection combined with the DET preprocessing method produced the most accurate MDA prediction model, having *R*^2^ and RMSE values for the calibration set of 0.66 and 2.28%, respectively, and the RPD of DET-sMC was 1.51.

**Table 1:** The calibration and validation results of PLS models for the prediction of MDA content in pine seedlings based on different spectral processing and variable selection methods.

		**Calibration**				**Validation**				**RPD**
		**R** ^**2**^		**RMSE**		**R** ^**2**^		**RMSE**		
**Pro-processing**	**Variable selection**	**Mean**	**SD**	**Mean**	**SD**	**Mean**	**SD**	**Mean**	**SD**	
OG	raw	0.64	0.04	2.31	0.12	0.61	0.00	2.97	0.00	1.30
	ga_sel	0.64	0.04	2.31	0.12	0.61	0.01	2.96	0.03	1.26
	rep_sel	0.64	0.04	2.31	0.12	0.61	0.00	2.97	0.00	1.33
	bve_sel	0.63	0.06	2.33	0.17	0.61	0.01	2.98	0.03	1.05
	sMC_sel	0.64	0.04	2.31	0.12	0.62	0.01	2.96	0.05	1.10
SNV	raw	0.65	0.01	2.28	0.03	0.61	0.00	2.98	0.01	1.42
	ga_sel	0.65	0.01	2.29	0.03	0.61	0.00	2.98	0.01	1.46
	rep_sel	0.65	0.01	2.28	0.03	0.61	0.00	2.97	0.00	1.40
	bve_sel	0.64	0.02	2.30	0.07	0.61	0.01	2.98	0.03	1.32
	sMC_sel	0.65	0.00	2.28	0.01	0.61	0.00	2.98	0.01	1.44
BS	raw	0.64	0.05	2.32	0.14	0.61	0.00	2.97	0.01	1.05
	ga_sel	0.64	0.04	2.32	0.13	0.61	0.00	2.97	0.01	1.06
	rep_sel	0.64	0.05	2.32	0.14	0.61	0.00	2.97	0.01	1.05
	bve_sel	0.64	0.05	2.32	0.15	0.61	0.00	2.98	0.02	1.04
	sMC_sel	0.64	0.02	2.30	0.07	0.61	0.01	2.96	0.03	1.50
BN	raw	0.64	0.04	2.31	0.12	0.61	0.00	2.97	0.00	1.30
	ga_sel	0.64	0.04	2.31	0.12	0.61	0.01	2.96	0.03	1.26
	rep_sel	0.64	0.04	2.31	0.12	0.61	0.00	2.97	0.00	1.33
	bve_sel	0.63	0.06	2.33	0.17	0.61	0.01	2.98	0.03	1.05
	sMC_sel	0.64	0.04	2.31	0.12	0.62	0.01	2.96	0.05	1.10
DET	raw	0.65	0.00	2.28	0.01	0.61	0.00	2.97	0.01	1.40
	ga_sel	0.65	0.01	2.28	0.02	0.61	0.00	2.97	0.00	1.48
	rep_sel	0.65	0.00	2.28	0.00	0.61	0.00	2.97	0.00	1.40
	bve_sel	0.65	0.01	2.28	0.02	0.61	0.02	2.99	0.06	1.35
	sMC_sel	0.66	0.00	2.28	0.00	0.61	0.00	2.97	0.00	1.51
BS_SNV	raw	0.65	0.01	2.28	0.02	0.61	0.00	2.97	0.02	1.40
	ga_sel	0.65	0.01	2.28	0.02	0.61	0.00	2.97	0.01	1.48
	rep_sel	0.65	0.01	2.28	0.02	0.61	0.01	2.97	0.02	1.40
	bve_sel	0.64	0.02	2.30	0.07	0.61	0.00	2.97	0.00	1.35
	sMC_sel	0.65	0.01	2.28	0.02	0.61	0.01	2.97	0.02	1.41
DET_SNV	raw	0.65	0.00	2.28	0.01	0.61	0.00	2.97	0.01	1.38
	ga_sel	0.65	0.01	2.28	0.02	0.61	0.00	2.97	0.00	1.33
	rep_sel	0.64	0.00	2.28	0.00	0.61	0.00	2.97	0.00	1.41
	bve_sel	0.65	0.01	2.29	0.02	0.61	0.02	2.99	0.06	1.19
	sMC_sel	0.65	0.00	2.29	0.00	0.61	0.00	2.97	0.00	1.43

*PLS, partial least squares; MDA, malondialdehyde; R^2^, correlation coefficient; RMSE, root mean square error; RPD, residual prediction deviation; SNV, standard normal variate; BS, block scale; BN, block normal; DET, detrended variable; BS-SNV, block scale and standard normal variate; DET-SNV, detrended variable and standard normal variate; ga, genetic algorithm; rep, regularization elimination; bve, inverse variable elimination; sMC, significant multivariate correlation*.

### Establishment of MDA Content Prediction Model Based on PLS

**Figure 1:**
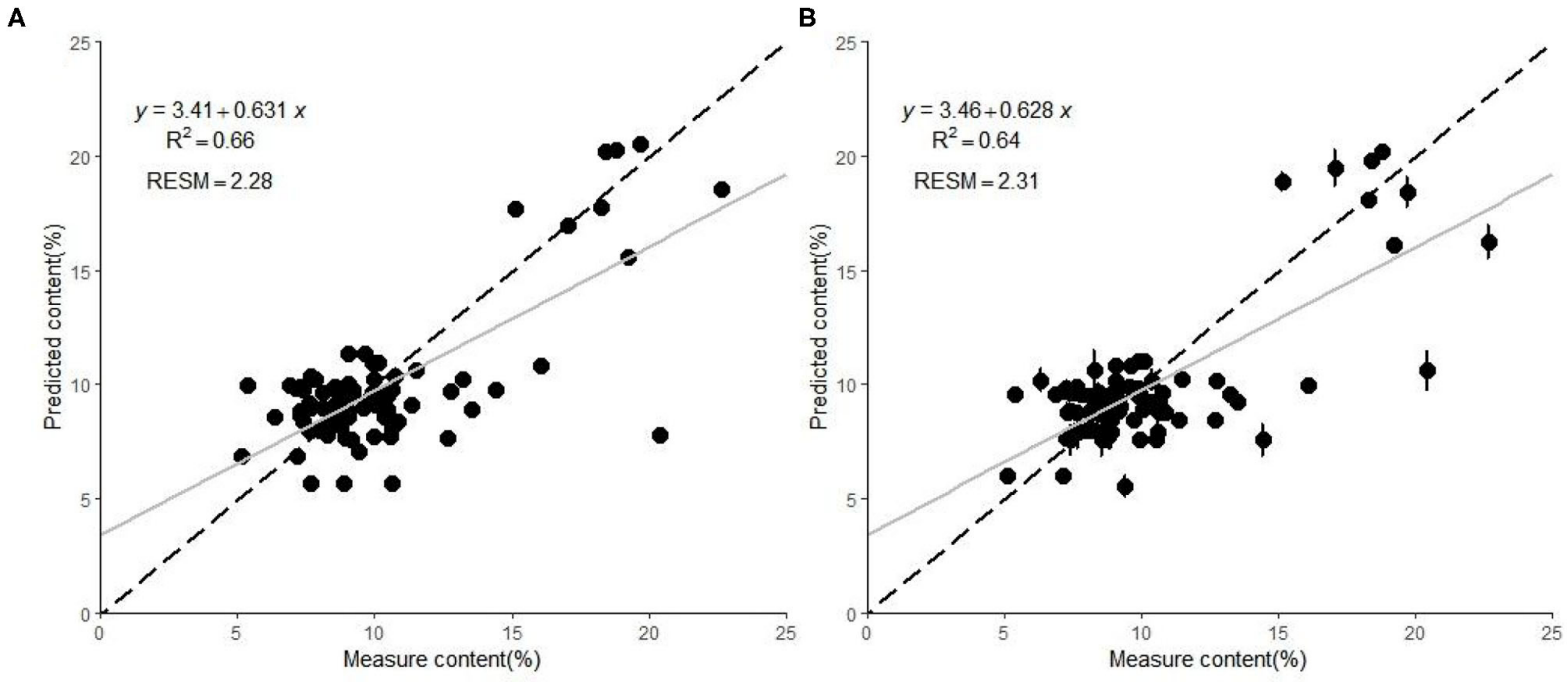
Scatterplots of the malondialdehyde (MDA) in pine seedlings predicted by **(A)** the detrended variable–significant multivariate correlation algorithm (DET-sMC) partial least squares (PLS) model and **(B)** the full-spectrum PLS model. The error bar of each symbol point represents the sample prediction error obtained by 100 random calibration models. The regression line of the model appears in gray; the dotted black line denotes the equality of measured vs. predicted MDA values.

**Figure 2:**
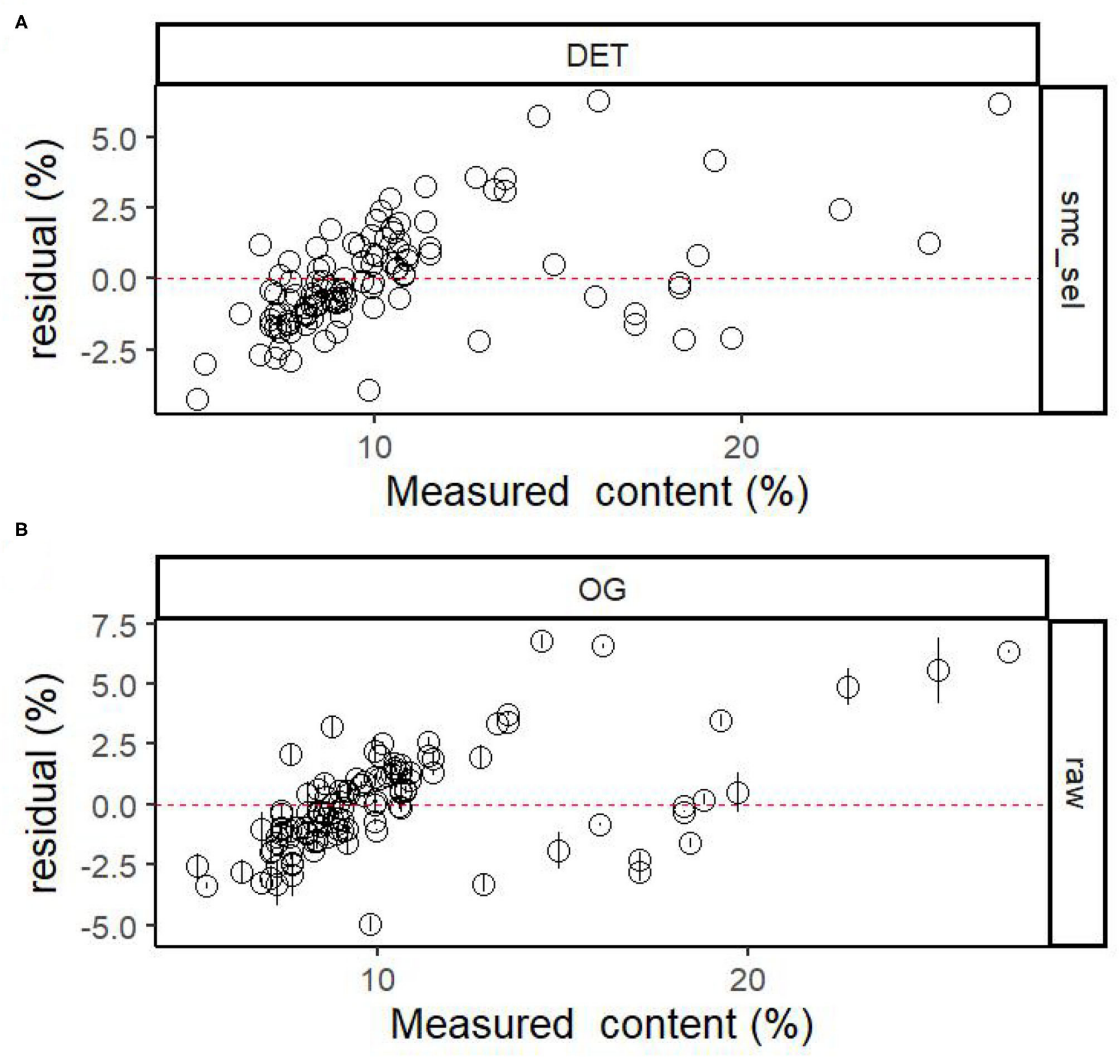
Residuals plotted against the measured MDA in pine seedlings based on **(A)** DET-sMC spectra and **(B)** full length of spectra. Error bars for predicted values represent the SDs obtained from the 100 simulated models.

The relationships between the MDA content predicted and measured by the PLS model when using the DET-sMC spectra and original full spectra are plotted in Figure 1. Although the prediction accuracy of each model differed, their prediction error was still low. The fitting model accuracy after spectral processing is slightly better than that based on original full spectra with only <10% of the full length of spectra. Figure 2 shows the residuals of the best processing spectral model for the MDA content. The latter tends to be underestimated when the measurement value is small. As the MDA content value increases, the predicted MDA value is more likely to be overpredicted. The residual values of the MDA model are all between −5 and 5%. In contrast, for the model established without any spectral preprocessing, the residual values of the MDA model are all between −5 and 7.5%.

**Figure 3:**
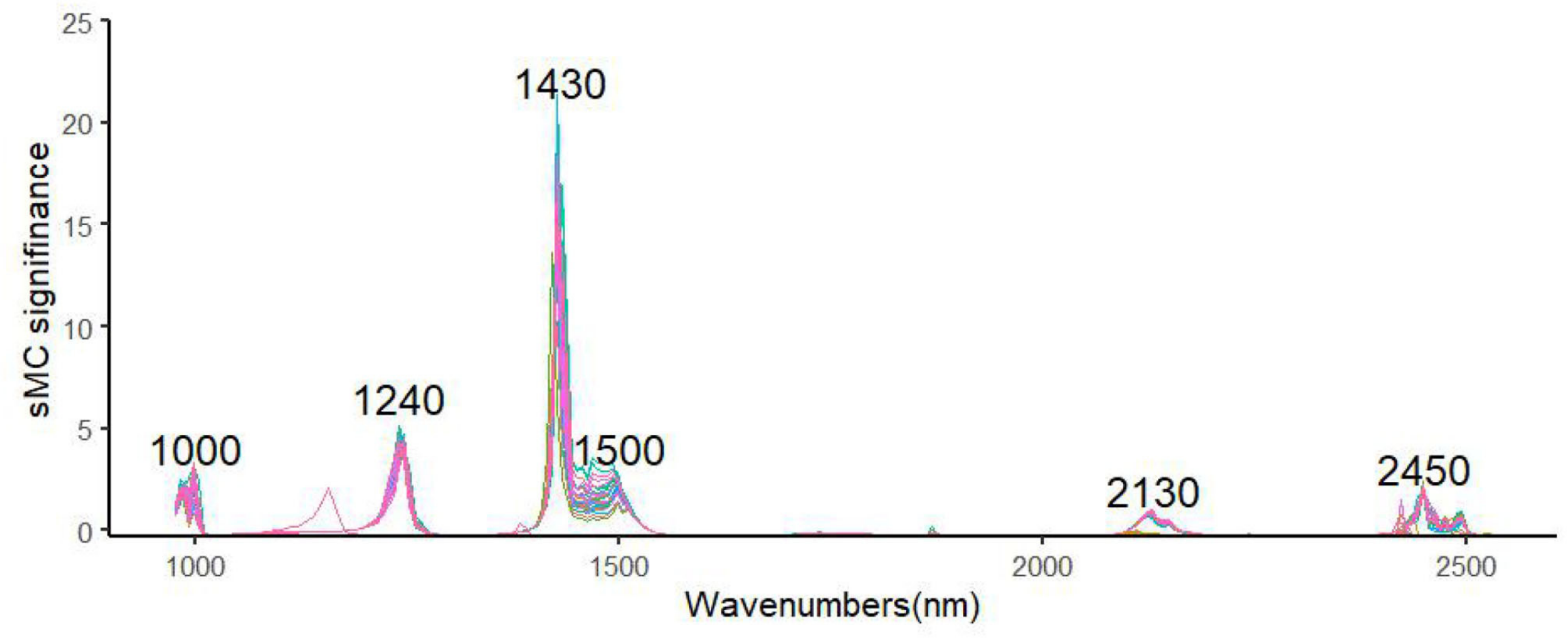
Spectral influence in the MDA models that were randomly run 100 times.

The important variable that was selected by using the sMC variable selection method 100 times in the MDA model on DET-sMC has exhibited in Figure 3. Evidently, as selected by 100 random models, the band selected by sMC is stable and robust, featuring several relative spectral regions in the prediction model. The variables at 1,000, 1,240, 1,430, 1,500, 2,130, and 2,450 nm were thus critical for building the MDA prediction model.

### MDA Variation Under Drought Stress Using the PLS Model

**Figure 4:**
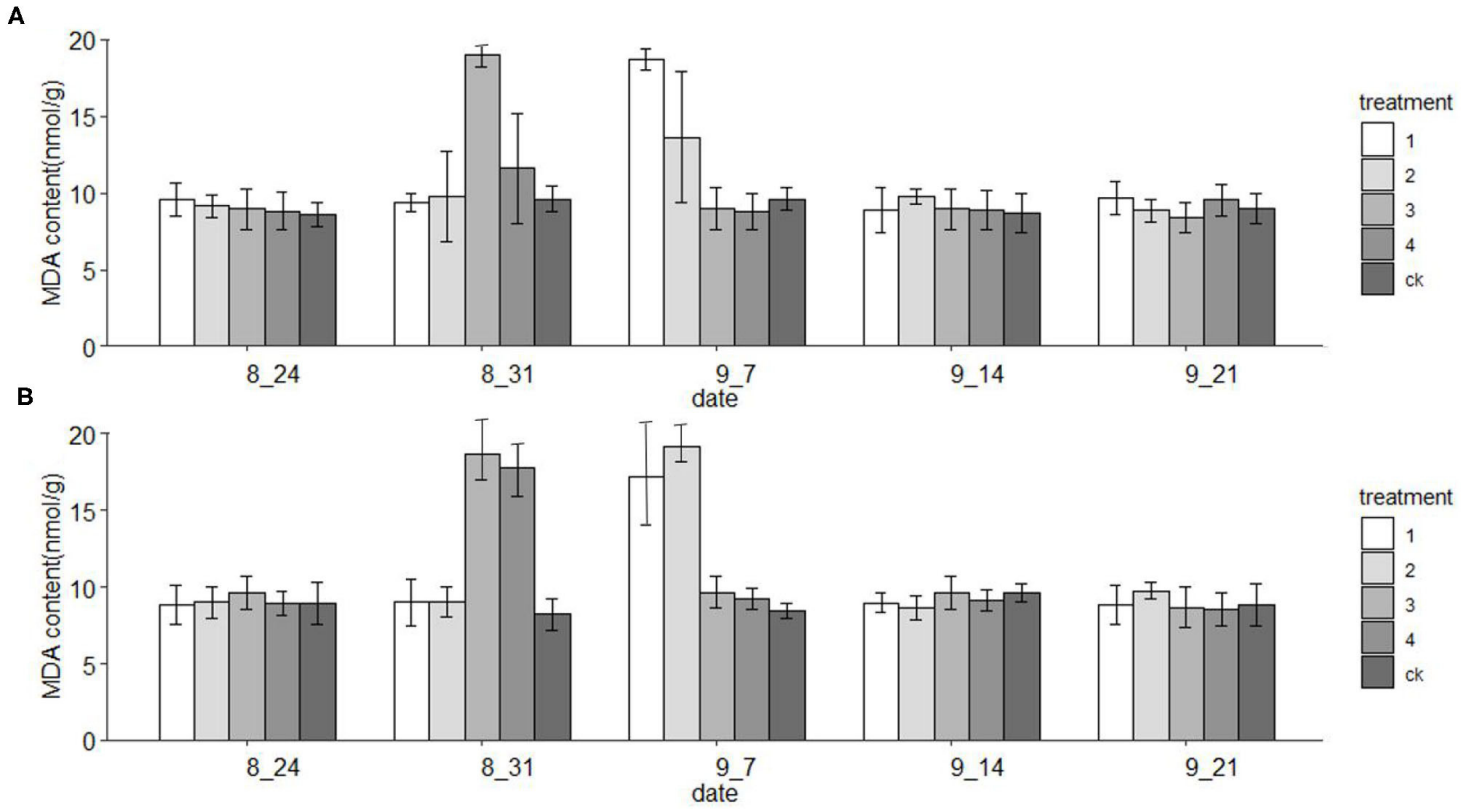
PLS model inversion of MDA composition in **(A)** loblolly pine and **(B)** slash pine.

The DET-sMC model was used to predict the MDA content of loblolly and slash pine seedlings under the same treatment. With more days elapsed since the initiation of each treatment, the MDA content of each treatment changed, by first increasing and then decreasing. In the two pine species, one-way ANOVA showed that on day 7 of drought exposure, the treatments entailing a 60% and 80% reduction in watering volume differed significantly from the other treatments (Figure 4). However, on day 14, those treatments with a 20 and 40% reduction in watering volume were significantly different from the other treatments. At other time points, the differences among treatments were not significant (Figure 4).

## Discussion

The purpose of this study was to reveal the applicability of NIR for the detection of MDA in loblolly pine and slash pine. The balance among production, elimination, and signal transduction of MDA is an important feature in redox biology and may determine the survival of plants under stress; hence, both enzymatic and non-enzymatic peroxidation processes can foster the formation of MDA and other oxidative peroxidation products in plants (Esterbauer et al., 1991; Morales and Munné-Bosch, 2019). Consequently, a quick and non-destructive method by which to monitor, *in situ*, the MDA content in plants under drought stress is a logistical priority, because it can enable timely and effective interventions by tree or crop managers to restore normal physiological activity. In this study, the relationship between MDA and NIR spectra was explored using seven spectral preprocessing methods and five effective variable selection algorithms. Comparing the results in Table 1, the DET preprocessing method coupled with the sMC variable selection method emerged as the optimal prediction model, with *R*^2^ and RMSE of 0.66 and 2.28%, respectively. These values were lower than those reported for predicting the MDA concentration (*R*^2^ = 0.996, RMSE = 2.117) in oilseed rape (*Brassica napus*) fresh leaves (Kong et al., 2016). The reason for this disparity may be leaf morphology. Oilseed rape plants have broad leaves, making the collection of their spectral data easier than for needles of slash or loblolly pine, whose single-leaf surface of a needle is very small and could not always be accurately positioned. This could have introduced relatively more irrelevant information in the collected spectral data, which reduced the modeling accuracy of the MDA content.

One advantage of using NIR spectroscopy is that it can model properties robustly without depending on unique chemical signals by applying statistical methods of chemometrics. Nonetheless, while collecting the NIR spectroscopy data, often high-frequency sounds, personnel operations, the external environment, and irrelevant noises are liable to interfere with this process, so it is vital to select effective spectral information (Guo et al., 2020). Accordingly, implementing appropriate spectral preprocessing and variable selection can improve the accuracy of a fitted model (Zou et al., 2010; Gerretzen et al., 2016). For example, the SNV-SVR model is based on SNV, which reduces particle size noise effects by scaling each spectrum to have an SD of 1.0 and by utilizing accurate prediction performance of both catechins and caffeine as suggested by Wang et al. (Wang et al., 2020); DET can reduce the curvature of each spectrum (Murphy et al., 2021); and BS can balance the effect of the modeled blocks, to avoid any block dominating the model (Mishra et al., 2021). In this study, this exercise shows that the sMC algorithm combined with PLS can efficiently identify the useful informative wavelength to provide a promising and robust correction model for the prediction of MDA. This finding is similar to another study that applied the sMC algorithm to get a robust model for predicting the chlorophyll content of *Sassafras tzumu* (Li et al., 2019). Several important variables that are related to MDA were selected similarly in each model, including those at regions corresponding to 1,000, 1,240, 1,430, 1,500, 2,130, and 2,450 nm. As reported by Kokaly et al., the regions around 1,500–1,600 nm are mainly related to O–H stretching vibration of aldehyde and phenolic compounds (Kokaly and Skidmore, 2015). Residual values are uniformly distributed in the horizontal band, which suggests that the selected model is more suitable, and coupled with a narrower bandwidth, which indicates a better fitting accuracy and implies greater accuracy of the fitted regression equation (Couture et al., 2016). Therefore, in this study, after DET-sMC processing of the spectral data, the range of residual values is smaller, and the model is more accurate.

For a comparative study, we then performed additional experiments for the inversion of the PLS model on the same treated loblolly and slash pine seedlings. In the prediction model results for this experiment, the MDA content increased at first but then decreased, a pattern consistent with the findings reported by Zhu (2014) and Wang et al. (2016), who, respectively, studied the physiological effects of water stress on *Pinus sylvestris* and *Larix gmelinii*. This shows that our optimal PLS model, which can detect the MDA content faster than *via* laboratory measurements, has a certain level of accuracy. However, the changes to the MDA content under the low-stress condition (i.e., treatments 1 and 2) and the high-stress condition (i.e., treatments 3 and 4) were not synchronous. The MDA content reached the maximum on day 7 under high-stress treatments but later on day 14 under the low-stress treatments (Figure 4). Previous studies have shown that the activity of lipoxygenase increase may lead to the formation of MDA and other lipid peroxidation products in plants, and that temperature is a critical factor influencing enzyme activity. Furthermore, our experiment was carried out in the hot summer, so that lipoxygenase activity was high, which may have led to the high MDA content detected under low drought stress. Therefore, membrane lipid peroxidation peaked earlier under high drought stress condition than under low stress condition, and seedlings were then adjusted to the drought environment. Similarly, with prolonged stress exposure, the MDA content also increased under high temperature and low-stress conditions.

## Conclusion

Near-infrared spectroscopy combined with PLS modeling provides a reliable and non-destructive way to predict the MDA content of pine trees. Furthermore, the spectral preprocessing methods and variable selection can successfully increase the model prediction accuracy with less variables used. Most importantly, we successfully applied this technology to predict MDA under drought stress in a fast and non-destructive way, thus demonstrating that the NIR-based technique harbors promising prospects for use in future studies of plant stress, especially for trees.

## Data Availability Statement

The original contributions presented in the study are included in the article/supplementary material, further inquiries can be directed to the corresponding author/s.

## Author Contributions

YZ conducted the experiment and wrote the manuscript. YL designed this study, supervised experiments, and performed revisions of the manuscript. QL supervised experiments and performed revisions of the manuscript. JJ supported the data collection and field experiment. All authors read and approved the final manuscript.

## Funding

This study was supported by the Fundamental Research Funds of CAF (CAFYBB2020SY008), the National Natural Science Foundation of China (31901323), Fundamental Research Funds of Chinese Forestry Academy (CAFYBB2017ZA001-2-1), and Zhejiang Science and Technology Major Program on Agricultural New Variety Breeding (2021C02070-8).

## Conflict of Interest

The authors declare that the research was conducted in the absence of any commercial or financial relationships that could be construed as a potential conflict of interest.

## Publisher's Note

All claims expressed in this article are solely those of the authors and do not necessarily represent those of their affiliated organizations, or those of the publisher, the editors and the reviewers. Any product that may be evaluated in this article, or claim that may be made by its manufacturer, is not guaranteed or endorsed by the publisher.
